# Tau PET imaging in Black and Latino participants with and without cognitive impairment

**DOI:** 10.1002/alz70856_106309

**Published:** 2026-01-09

**Authors:** Gregory S Day, Scott A. Przybelski, Manoj K Jain, Christian Lachner, Yoav D Piura, Paula Aduen, Leah Schecter, Angela J Fought, Patricia Diaz‐Galvan, Val J Lowe, Christopher G Schwarz, Brad F Boeve, Ronald Petersen, Kejal Kantarci, Neill R. Graff‐Radford

**Affiliations:** ^1^ Mayo Clinic in Florida, Jacksonville, FL, USA; ^2^ Mayo Clinic, Rochester, MN, USA; ^3^ Department of Radiology, Mayo Clinic, Rochester, MN, USA

## Abstract

**Background:**

Tau‐PET neuroimaging is increasingly applied to diagnose and stage, track progression, and assess response to putative disease‐modifying treatments in individuals with Alzheimer disease. However, it remains to be seen whether findings established in predominantly non‐Hispanic White (nHW) cohorts will generalize to Black and Latino participants who are underrepresented in dementia research.

**Method:**

Community‐dwelling Black (*n* = 83) and Latino participants (*n* = 27) completed MRI (with quantification of white matter hyperintensity volumes), and amyloid‐ (PiB or florbetapir) and tau‐ (flortaucipir) PET brain imaging as part of a longitudinal study of memory and aging at Mayo Clinic in Florida. Global and regional tau‐PET standardized uptake value ratios (normalized to the cerebellar crus) were determined in Black and Latino participants and compared with findings from an independent cohort of nHW participants (*n* = 110), 1:1‐matched for age, sex, education, and global Clinical Dementia Rating®. Conditional logistic modeling compared 47 regions of interest, adjusted for false discovery rate. Relevant associations between flortaucipir retention and clinical outcomes were assessed using Spearman rank correlations.

**Result:**

Eighty‐three Black (mean 72±10.8‐years‐old, 63% female, 33% cognitively impaired) and 27 Latino participants (mean 65.8‐years‐old, 56% female, 11% cognitively impaired) completed neuroimaging. Data were compared with findings in 110 matched nHW individuals. Global tau‐PET burden was similar between matched cohorts (Black and nHW participants, mean SUVR: 1.34±0.39 vs 1.36±0.37, *p* = 0.71; Latino and nHW participants, mean SUVR: 1.17±0.09 vs 1.20±0.11, *p* = 0.66). Lower flortaucipir uptake was noted in the caudate (*p* = 0.005) and putamen (*p* = 0.022) of Black/Latino participants (vs nHW), although differences attenuated following adjustment for white matter hyperintensity volume. Global flortaucipir retention increased with age (Black: Rho=0.31; Latino: Rho=0.46; nHW: Rho=0.27; *p* <0.05), amyloid Centiloids (Black: Rho=0.65; Latino: Rho=0.41; nHW: Rho=0.58; *p* <0.05), and cognitive impairment (i.e., Clinical Dementia Rating® Sum‐of‐Box scores, Black/Latino: Rho=0.31, *p* = 0.07; nHW: 0.76, *p* <0.001).

**Conclusion:**

Tau‐PET patterns were similar across ethnoracial groups after adjusting for white matter hyperintensity volumes—a biomarker of small vessel disease. Flortaucipir retention increased with age, amyloid accumulation, and cognitive impairment across all cohorts. These findings affirm the clinical and research applications of tau PET in diverse cohorts.

**Acknowledgements**: Flortaucipir precursor and technology was supported by Avid Radiopharmaceuticals.

